# Cerebrospinal fluid α-synuclein adds the risk of cognitive decline and is associated with tau pathology among non-demented older adults

**DOI:** 10.1186/s13195-024-01463-2

**Published:** 2024-05-10

**Authors:** Wenying Liu, Wenwen Li, Zhaojun Liu, Yan Li, Xuechu Wang, Mengmeng Guo, Shiyuan Wang, Shuheng Wang, Yan Li, Jianping Jia

**Affiliations:** 1 Innovation Center for Neurological Disorders and Department of Neurology, National Clinical Research Center for Geriatric Diseases, Xuanwu Hospital, Capital Medical University, Beijing, 100053 China; 2 The National Clinical Research Center for Mental Disorders & Beijing Key Laboratory of Mental Disorders, Beijing Anding Hospital, Capital Medical University, Beijing, China; 3 Advanced Innovation Center for Human Brain Protection, Capital Medical University, Beijing, China; 4 Beijing Key Laboratory of Geriatric Cognitive Disorders, Beijing, 100053 China; 5 Clinical Center for Neurodegenerative Disease and Memory Impairment, Capital Medical University, Beijing, 100053 China; 6 Center of Alzheimer’s Disease, Collaborative Innovation Center for Brain Disorders, Beijing Institute of Brain Disorders, Capital Medical University, Beijing, 100053 China; 7 Key Laboratory of Neurodegenerative Diseases, Ministry of Education, Beijing, 100053 China

**Keywords:** Dementia, α-synuclein, Tau, Inflammation

## Abstract

**Background:**

The role of α-synuclein in dementia has been recognized, yet its exact influence on cognitive decline in non-demented older adults is still not fully understood.

**Methods:**

A total of 331 non-demented individuals were included in the study from the Alzheimer’s Disease Neuroimaging Initiative (ADNI). Participants were divided into two distinct groups based on their α-synuclein levels: one with lower levels (α-synuclein-L) and another with higher levels (α-synuclein-H). Measurements included neuropsychiatric scales, cerebrospinal fluid (CSF) biomarkers, and blood transcriptomics. The linear mixed-effects model investigated the longitudinal changes in cognition. Kaplan-Meier survival analysis and the Cox proportional hazards model were utilized to evaluate the effects of different levels of α-synuclein on dementia. Gene set enrichment analysis (GSEA) was utilized to investigate the biological pathways related to cognitive impairment. Pearson correlation, multiple linear regression models, and mediation analysis were employed to investigate the relationship between α-synuclein and neurodegenerative biomarkers, and their potential mechanisms affecting cognition.

**Results:**

Higher CSF α-synuclein levels were associated with increased risk of cognitive decline and progression to dementia. Enrichment analysis highlighted the activation of tau-associated and immune response pathways in the α-synuclein-H group. Further correlation and regression analysis indicated that the CSF α-synuclein levels were positively correlated with CSF total tau (t-tau), phosphorylated tau (p-tau) 181, tumor necrosis factor receptor 1 (TNFR1) and intercellular cell adhesion molecule-1 (ICAM-1). Mediation analysis further elucidated that the detrimental effects of CSF α-synuclein on cognition were primarily mediated through CSF t-tau and p-tau. Additionally, it was observed that CSF α-synuclein influenced CSF t-tau and p-tau181 levels via inflammatory pathways involving CSF TNFR1 and ICAM-1.

**Conclusions:**

These findings elucidate a significant connection between elevated levels of CSF α-synuclein and the progression of cognitive decline, highlighting the critical roles of activated inflammatory pathways and tau pathology in this association. They underscore the importance of monitoring CSF α-synuclein levels as a promising biomarker for identifying individuals at increased risk of cognitive deterioration and developing dementia.

**Supplementary Information:**

The online version contains supplementary material available at 10.1186/s13195-024-01463-2.

## Background

α-synuclein is abundantly present in the brain, notably within neurons, especially at synaptic terminals, where it plays a crucial role in vesicle transport and neurotransmitter release [1]. In addition to its presence in the brain, small oligomeric aggregates of α-synuclein can also be found in the blood and cerebrospinal fluid (CSF) [2]. α-synuclein aggregates in the cytoplasm of neurons lead to the formation of Lewy bodies, which are largely identified in dementia and neurodegenerative diseases. The progressive accumulation of α-synuclein at the presynaptic terminal is strongly linked to the pathogenesis of Dementia with Lewy Bodies (DLB) [3]. Early disruptions caused by α-synuclein oligomers in the striatal cholinergic system are believed to trigger cognitive and motor impairments at the onset of Parkinson’s disease (PD) [4]. Although Alzheimer’s disease (AD) is primarily characterized by amyloid-β (Aβ) plaques and tau neurofibrillary tangles [5], it seldom presents in isolation. In fact, co-pathology is common, with 30%–50% of AD cases also showing signs of α-synuclein pathology [6].

Although α-synuclein has been identified in various types of dementia, the potential role of CSF α-synuclein in dementia progression is not fully understood, with studies yielding conflicting results regarding its impact on cognition. Some studies have indicated that patients with impaired cognition exhibit increased levels of α-synuclein in their CSF compared to healthy controls [7]. However, a case–control study involving 90 participants found no significant differences in CSF α-synuclein levels between individuals with dementia and healthy controls [8]. Additionally, another study reports that AD patients with mini-mental state examination (MMSE) scores below 20 have significantly lower levels of α-synuclein compared to AD patients with MMSE scores of 20 or above [9].

Discrepancies in findings regarding the role of α-synuclein in cognitive decline might be attributed to the co-exist of other pathological proteins, such as total tau (t-tau), phosphorylated tau (p-tau), amyloid-beta (Aβ), and inflammatory markers, all of which are associated with cognitive decline. There is a suggested interdependency among these proteins in neurodegeneration [10]. Experimental research indicates that α-synuclein, p-tau, and Aβ42 act synergistically, accelerating their accumulation and leading to rapid cognitive decline in transgenic DLB-AD mice [11]. Clinical research suggests that the regional overlap of α-synuclein and tau may promote mutual propagation, contributing to a more rapid disease progression [12–14]. In addition, in-vivo studies indicated that α-synuclein could induce immune response and accelerate neurodegeneration by regulating inflammatory molecules, such intercellular adhesion molecule 1 (ICAM-1) and tumor necrosis factor receptor (TNFRs) [15, 16]. These molecular interactions suggest a complex mechanism potentially affecting cognition, making it unclear whether α-synuclein directly impacts cognition or does so indirectly through other pathological proteins. Yet, research exploring the impact of α-synuclein, along with other neurodegenerative factors, in a broader population of non-demented older adults remains scarce.

This study aims to investigate the association between CSF α-synuclein levels and cognitive impairment, utilizing longitudinal data from the Alzheimer’s Disease Neuroimaging Initiative (ADNI) database. It involves comprehensive assessments including neuropsychiatric scales, CSF biomarkers, and blood transcriptomics. Through correlation analyses and Gene set enrichment analysis (GSEA), the study aims to unravel the connections among CSF biomarkers and cognitive impairment. This study aims to offer new insights into the role of α-synuclein in dementia, addressing current gaps in understanding and exploring the mechanisms that underlie cognitive deterioration.

## Methods

### Study participants

**Fig. 1:**
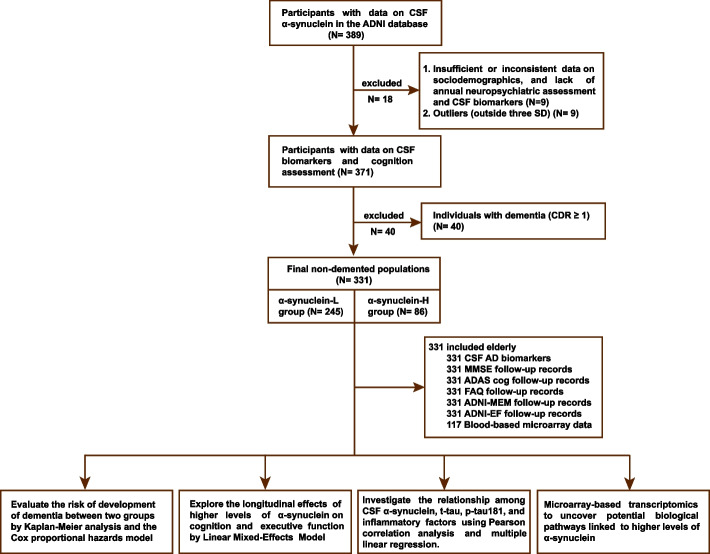
Flowchart in this study

This study recruited 331 individuals aged between 55 and 90 from the ADNI database (adni.loni.usc.edu), including participants who were either cognitively unimpaired (CU) or had mild cognitive impairment (MCI). The ADNI, a multicenter, longitudinal study, focuses on the exploration of AD through clinical features, imaging, genetic, and biochemical biomarkers. Ethical approval for this research was obtained from the institutional review boards of all involved institutions, and written informed consent was secured from every participant. Each participant was subjected to CSF biomarker detection and a detailed in-person neuropsychological assessment at baseline, followed by annual follow-ups. Individuals with α-synuclein levels exceeding 3 standard deviations (SD) from the mean, diagnosed with dementia at baseline, or lacking annual neuropsychiatric assessments were excluded from the study (refer to Fig. 1). The surv_cutpoint function from the R package survminer identified 0.68 ng/mL as the optimal cut-off value for α-synuclein as an indicator of dementia risk (see Additional file 1, Fig. S1). Accordingly, participants were divided into a lower α-synuclein group (α-synuclein-L, *n* = 245) and a higher α-synuclein group (α-synuclein-H, *n* = 86). Detailed information on the inclusion and exclusion criteria is available in Additional file 1.

### Apolipoprotein E type 4 allele (*APOE* ε4) genotype

DNA extraction was performed using the QIAamp® DNA Blood Mini Kit, followed by amplification through polymerase chain reaction (PCR). This process utilized forward primers 5′-ACG GCT GTC CAA GGA GCT G-3′ for rs429358 and 5′-CTC CGC GAT GCC GAT GAC-3′ for rs7412. Following this, the *APOE* ε4 genotype determination was carried out employing restriction fragment length polymorphism analysis.

### Measurements of α-synuclein, AD biomarkers, and inflammatory cytokines

The measurements of CSF Aβ42, t-tau, and p-tau within the ADNI database were conducted by the University of Pennsylvania’s ADNI Biomarker Core. These measurements employed the multiplex xMAP Luminex platform (Luminex Corp, Austin, TX, USA) in conjunction with the INNOBIA AlzBio3 assay kit (Fujirebio, Ghent, Belgium). For the determination of total CSF α-synuclein levels, which includes both soluble and aggregated forms, the Luminex MicroPlex Microspheres (Luminex Corp, Austin, TX) were used, alongside a biotinylated goat anti-human α-synuclein antibody provided by R&D Systems (catalog # BAF1338). Additionally, the concentrations ofTNFR1 and ICAM-1 were quantified at the William Hu lab at Emory University using ELISA kits. To ensure the accuracy and consistency of the results, all samples, along with six CSF standards per plate, were processed in duplicate and normalized against the values obtained from the CSF standards.

### Blood‑based microarray profiling and analysis

RNA was extracted from whole blood samples, which were collected in PAXgene Blood RNA Tubes, using the PAXgene Blood RNA Kit by Qiagen Inc., Valencia, CA, USA. In the ADNI study, gene expression profiling was carried out using the Affymetrix Human Genome U219 Array (Affymetrix, Santa Clara, CA, USA). The quality of gene expression data, including aspects like sample integrity, hybridization efficiency, and overall signal strength, was evaluated using the Affymetrix Expression Console software in conjunction with Partek Genomic Suite 6.6. The raw expression data were then processed using the robust multichip average (RMA) method for normalization.

To explore the pathways associated with cognitive decline, enrichment analysis was conducted on 89 participants in the α-synuclein-L group and 28 participants in the α-synuclein-H group. For this analysis, GSEA was employed, utilizing a total of 20,092 expressed genes. The GSEA, executed in R, referenced the biological process (BP) category of the Gene Ontology (GO) terms. Pathway analysis between the groups was performed using the Disease-Perturbations-from-GEO-up.txt file from the Enrichr database. In this analysis, an adjusted *p* value < 0.05 was considered to denote statistical significance.

### Statistical analysis

To assess the distribution normality of each biomarker, the Kolmogorov-Smirnov test was employed. For those variables not adhering to a normal distribution, a box-cox normalization procedure was applied to transform the data accordingly. The impact of α-synuclein levels on cognitive progression was examined using Kaplan-Meier survival analysis, with differences between groups evaluated through the log-rank test. Additionally, the Cox proportional hazards model was utilized to determine the risk associated with α-synuclein levels, taking into account potential confounders such as age, sex, education level, baseline MMSE, and *APOE* ε4 genotype. The influence of α-synuclein on longitudinal clinical outcomes, specifically cognitive and social activity functions, was investigated using a linear mixed-effects model. To explore the relationships between α-synuclein, inflammatory cytokines, and tau pathology, Pearson correlation tests were conducted, along with multiple linear regression analyses. These analyses were adjusted for age, sex, education, and *APOE* ε4 genotype to control for potential confounding factors. Mediation analysis was carried out to further understand the pathways through which α-synuclein may influence cognitive impairment. This analysis was adjusted for age, education, sex, and *APOE* ε4 genotype, employing the mediation package in R with 1000 bootstrap samples to estimate the average direct effects (ADE), average causal mediation effects (ACME), and mediation proportion of the variables involved. A *p*-value of less than 0.05 was considered statistically significant for all tests. All analyses were performed using SPSS 17.0 and R version 4.3.2.

## Results

### Baseline demographic and clinical characteristics of participants from the ADNI database

At baseline, this study involved a total of 331 individuals without dementia. It was observed that the CU group exhibited lower levels of α-synuclein, t-tau, and p-tau181, and higher levels of Aβ42 compared to MCI group (see Additional file 1: Table S1). Utilizing the surv_cutpoint function from the R package survminer, the optimal cut-off value for α-synuclein in predicting the risk of dementia was determined to be 0.68 ng/mL (see Additional file 1: Fig. S1). The baseline characteristics of the two groups, categorized by α-synuclein levels, are presented in Table 1. There were no differences in age, gender, and *APOE* ε4 genotype. Participants in the α-synuclein-H group demonstrated more pronounced cognitive impairment and decreased executive function compared to their counterparts. This group also showed elevated levels of CSF t-tau, p-tau181, and TNFR1, while CSF Aβ42 and ICAM-1 levels remained similar across both groups.

**Table 1:** Characteristics of participants in the ADNI dataset

Characteristics		α-synuclein-L group	α-synuclein-H group	*p* value
N		245	86	
Age (years)		74.60 (6.77	75.00 (7.03)	0.668^a^
Gender (female/male, %)		64.43	56.36	0.700^b^
Diagnosis	CU	89	15	0.002^b^
	MCI	156	71	
APOE4 (ε4 ratio, %)		44.49	54.16	0.346^b^
Education		15.70 (2.92)	15.50 (3.36)	0.636^a^
MMSE		27.40 (2.16)	26.70 (2.32)	0.015^a^
ADNI-MEM		0.18 (0.81)	-0.06 (0.75)	0.012^a^
ADAS-cog 11		10.40 (5.28)	11.80 (5.71)	0.038^a^
FAQ		3.10 (4.64)	4.71 (5.40)	0.003^a^
ADNI-EF		0.10 (0.91)	-0.25 (0.89)	0.003^a^
CSF biomarkers at baseline				
α-synuclein (ng/mL)		0.46 (0.46)	1.28 (0.69)	< 0.0001^a^
Aβ42 (pg/mL)		967.00 (534.00)	1034.00 (732.00)	0.810^a^
t-tau (pg/mL)		263.00 (92.80)	376.00 (124.00)	< 0.0001^a^
p-tau181 (pg/mL)		25.50 (10.70)	37.40 (14.20)	< 0.0001^a^
TNFR1 (pg/mL)		839.00 (181.00)	1003.00 (279.00)	< 0.0001^a^
ICAM-1 (ng/mL)		367.00 (193.00)	411.00 (222.00)	0.1020^a^

*Abbreviations: CU* Cognitively unimpaired, *MCI* Mild cognitive impairment, *APOE ε4* Apolipoprotein E type 4 allele, *MMSE*, Mini-Mental State Examination, *ADNI-MEM* Alzheimer’s Disease Neuroimaging Initiative memory score, *ADAS-cog 11* Alzheimer’s Disease Assessment Scale cognitive Sect. 11-item, *FAQ* Functional Activities Questionnaire, *ADNI-EF* Alzheimer’s Disease Neuroimaging Initiative executive function score, *CSF* Cerebrospinal Fluid, *Aβ42* Amyloid beta 42, *t-tau* Total tau, *p-tau181* Phosphorylated tau at threonine 181, *TNFR1*, Tumor necrosis factor receptor 1, *ICAM-1* Intercellular adhesion molecule 1

^a^Mann-Whitney U test

^b^Chi-squared test

### Longitudinal effects of different α-synuclein groups on cognitive decline

**Fig. 2:**
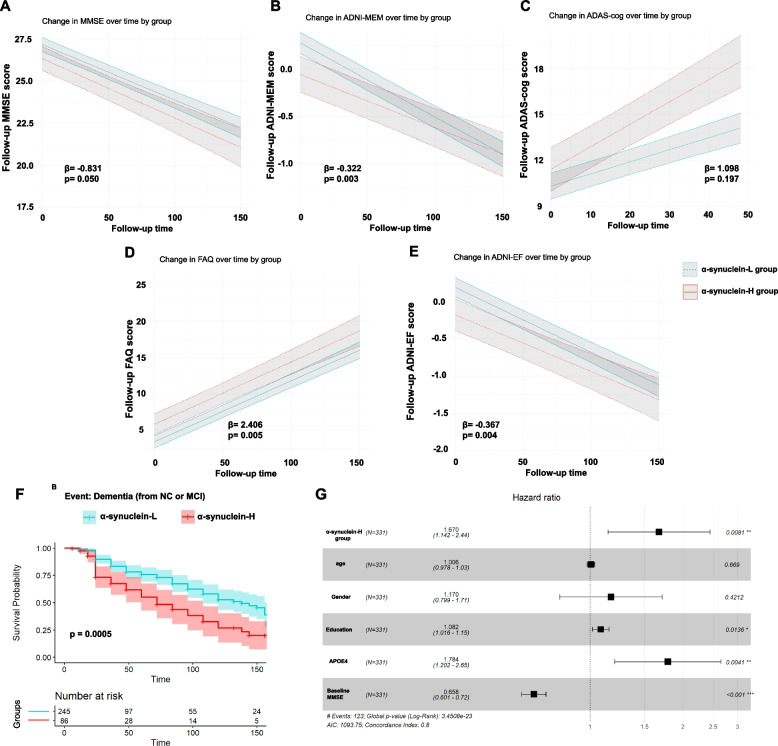
Longitudinal effects of different α-synuclein groups on cognitive decline. The α-synuclein-H group experienced more significant longitudinal cognitive decline and executive function impairment than those in the α-synuclein-L group. Specifically, significant declines were observed in the MMSE (**A**) and ADNI-MEM (**B**) scores, as well as in executive function as measured by the FAQ (**D**) and ADNI-EF (**E**) scores. There is a significant divergence in the rate of change during the follow-up in ADAS-cog (**C**). The α-synuclein-H group showing a higher rate of progression from CU or MCI to dementia (**F**). When accounting for age, gender, education, and *APOE* ε4 genotype, the Cox proportional hazards model further substantiated the elevated risk of cognitive progression in the α-synuclein-H group, compared to the α-synuclein-L group (**G**). Abbreviations: MMSE: Mini-Mental State Examination; ADNI-MEM: Alzheimer’s Disease Neuroimaging Initiative memory score; ADAS-cog: Alzheimer’s Disease Assessment Scale cognitive; FAQ: Functional Activities Questionnaire; ADNI-EF: Alzheimer’s Disease Neuroimaging Initiative executive function score; CU: cognitively unimpaired; MCI: mild cognitive impairment; *APOE* ε4: Apolipoprotein E type 4 allele

A total of 331 individuals participated in baseline and annual follow-up interviews, accumulating up to 150 months of follow-up time. Compared to those in the α-synuclein-L group, subjects in the α-synuclein-H group exhibited greater longitudinal cognitive decline (β = -0.831, *p* = 0.050 for MMSE; β = -0.322, *p* = 0.003 for ADNI-MEM; Fig. 2A and B) and executive function impairment (β = 2.406, *p* = 0.005 for FAQ; β = -0.367, *p* = 0.004 for ADNI-EF; Fig. 2D, E). There is a significant divergence in the rate of change during the follow-up in ADAS-cog (β = 0.068, *p* < 0.0001 for ADAS-cog; Fig. 2C and Additional file 2: Table S2).

During the follow-up period, within the α-synuclein-L group, there were 156 individuals who remained cognitively unimpaired, 89 with MCI, 12 who progressed from normal cognition to dementia, and 67 who transitioned from MCI to dementia. In the α-synuclein-H group, there were 15 individuals with normal cognition, 71 with MCI, 2 progressed from normal cognition to dementia, and 42 transitioned from MCI to dementia. Notably, a significantly higher conversion rate from CU or MCI to dementia was observed in the α-synuclein-H group (log-rank χ^2^ = 12.121, *p* = 0.0005, Fig. 2F). After adjusting for age, gender, education, and *APOE* ε4 genotype, individuals in the α-synuclein-H group were found to have an increased risk of cognitive progression to dementia compared to those in the lower group, as indicated by the Cox proportional hazards model (HR = 1.670, 95% CI: 1.142 to 2.440, *p* = 0.008, Fig. 2G). No significant differences were observed in the conversion patterns from non-Alzheimer’s to AD (according to the 2018 NIA-AA diagnostic criteria) between the two α-synuclein groups, suggesting a more complex relationship between α-synuclein levels and the specific pathways leading to AD (see Additional file 2: Fig. S2).

### Enrichment analysis in α-synuclein-H group and correlation analysis with CSF biomarkers

**Fig. 3:**
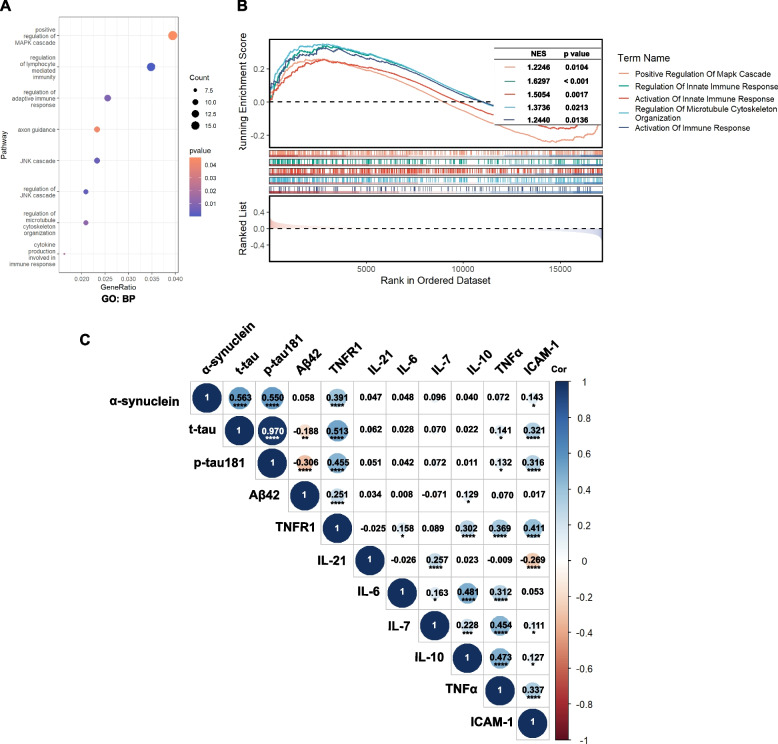
Enrichment analysis in α-synuclein-H group and correlation analysis with CSF biomarkers. The GO analysis pointed to a significant enrichment in pathways related to the tau protein, specifically noting the MAPK cascades and pathways involved in immune responses, predominantly in the α-synuclein-H group (**A**). Moreover, GSEA results also underscored an upregulation in MAPK cascades and immune response pathways (**B**). Pearson correlation analysis demonstrated significant associations among CSF α-synuclein, CSF t-tau, CSF p-tau181, CSF TNFR1, and CSF ICAM-1 (**C**). Abbreviations: BP: biological process; Gene set enrichment analysis: GSEA; CSF: Cerebrospinal Fluid; t-tau: Total tau; p-tau181: Phosphorylated tau at threonine 181; TNFR1: Tumor necrosis factor receptor 1; ICAM-1: Intercellular adhesion molecule 1; MAPK: Mitogen-activated protein kinase

Enrichment analysis was conducted on 89 α-synuclein-L and 28 α-synuclein-H subjects to explore pathways associated with cognitive decline. A total of 20,092 expressed genes were analyzed using GSEA. The GO BP analysis, utilizing the Enrichr database, identified significant enrichment in pathways related to tau protein and immune response pathways in individuals with elevated α-synuclein levels (Fig. 3A). The GSEA results also indicated an upregulation in the MAPK cascades and immune response pathways (Fig. 3B). Furthermore, Pearson correlation analysis demonstrated that α-synuclein was correlated with t-tau (*r* = 0.563, *p* < 0.0001), p-tau 181(*r* = 0.550, *p* < 0.0001), TNFR1 (*r* = 0.391, *p* < 0.0001) and ICAM-1 (*r* = 0.143, *p* = 0.011) (Fig. 3C). Moreover, both TNFR1 and ICAM-1 were found to be associated with t-tau and p-tau181 (Fig. 3C).

### Association of α-synuclein with CSF tau pathology and inflammatory cytokines

**Fig. 4:**
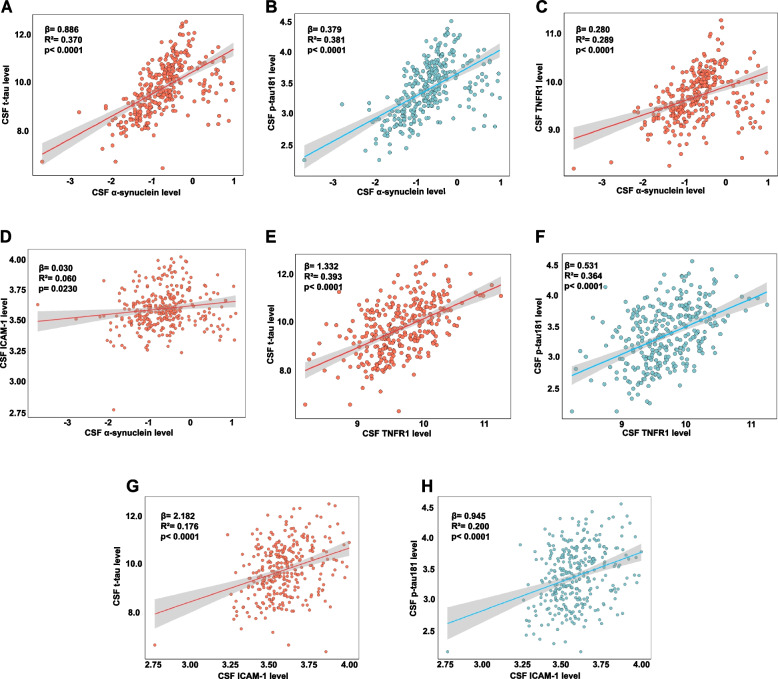
Association of α-synuclein with CSF tau pathology and inflammatory cytokines. The multiple linear regression model revealed that CSF α-synuclein showed a positive correlation with CSF t-tau, p-tau181, TNFR1, and ICAM-1, with adjusting for age, sex, education, and *APOE* ε4 genotype (**A**-**D**). Furthermore, CSF TNFR1 and ICAM-1 were also positively correlated with both CSF t-tau and p-tau181 (**E**–**H**)

To delve deeper into the relationship among CSF α-synuclein levels, tau pathology, and inflammatory cytokines, a multiple linear regression model was utilized, with adjustments made for age, sex, education, and *APOE* ε4 genotype. The analysis revealed that α-synuclein levels were positively correlated with t-tau (β = 0.886, *p* < 0.0001), p-tau (β = 0.379, *p* < 0.0001), TNFR1 (β = 0.280, *p* < 0.0001), and ICAM-1 (β = 0.030, *p* = 0.0230) (Fig. 4A-D). The above inflammatory factors also found to be positively correlated with both t-tau and p-tau181, indicating a complex network of interactions among these biomarkers related to neurodegeneration.

### Interaction and mediation analyses among CSF α-synuclein, tau pathology, inflammatory cytokines, and cognitive impairment

**Fig. 5:**
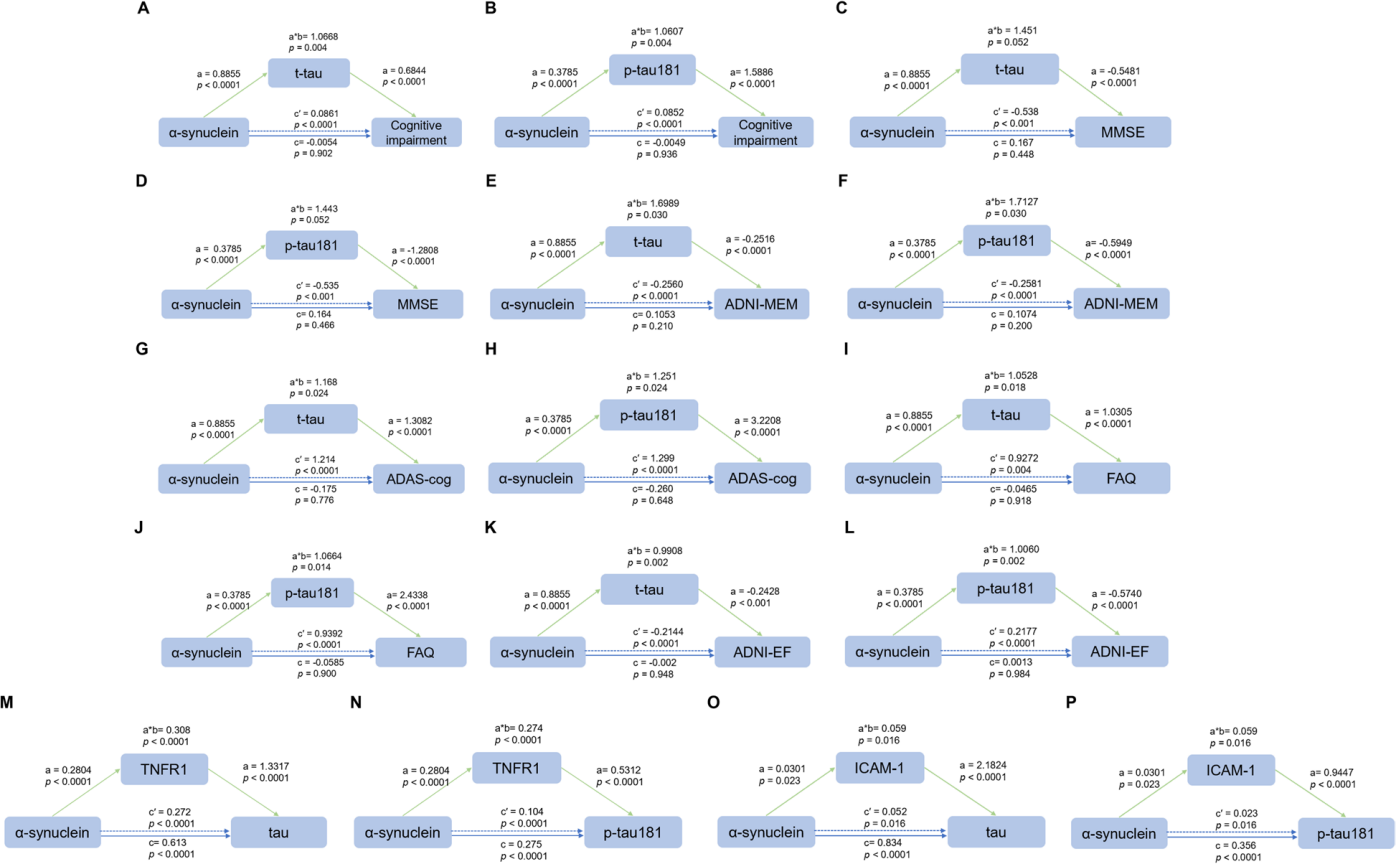
Interaction and mediation analyses among CSF α-synuclein, tau pathology, inflammatory cytokines, and cognitive impairment. The mediation analysis indicated that the ACME of α-synuclein on cognitive impairment and executive function were notably significant when the mediation was through both t-tau and p-tau181, with the mediation proportion also reaching significance **(A-L)**. The findings highlighted that both the ADE and ACME of α-synuclein on tau pathology via inflammatory pathways were significant (**M**-**P**). **Note:** The blue lines showed the ADE (c), the blue dotted lines showed the ACME (c′), and the a*b depicted the mediation effects. The path weight was expressed as an effect and a *p*-value. Abbreviations: ADE: average direct effects; ACME: average causal mediation effects

Further analysis was conducted to explore the contributions of CSF α-synuclein and inflammatory factors to tau pathology and cognitive impairment, utilizing mediation models and adjusting for age, gender, education, and *APOE* ε4 genotype. While the ADE of α-synuclein on cognitive impairment were not significant, notable ACME were observed when mediation occurred through either. The mediation proportion was also significant (a*b = 1.0668, *p* = 0.004 in Model A, mediated by t-tau; a*b = 1.0607, *p* = 0.004 in model B, mediated by p-tau181; Fig. 5A, B). This pattern of influence remained consistent across a variety of neuropsychiatric assessments, including the MMSE, ADNI-MEM, ADAS-cog, FAQ, and ADNI-EF (Fig. 5 C-L).

Additionally, a positive association was noted between α-synuclein levels and inflammatory markers (TNFR1 and ICAM-1), which were also positively correlated with both t-tau and p-tau181. The analysis indicated that the indirect effects and the proportion of mediation of α-synuclein’s impact on tau pathology via TNFRs and ICAM-1 were significant (Fig. 5M-P). These findings suggest that the impact of α-synuclein on cognitive and executive functions is primarily mediated through tau pathology, with α-synuclein also potentially exacerbating tau pathology through inflammatory pathways.

## Discussion

This study revealed that individuals with elevated levels of CSF α-synuclein were at a higher risk of converting to dementia compared to those with lower levels of α-synuclein. The potential biological mechanisms behind this association may involve the activation of tau-related and immune response pathways. CSF α-synuclein showed a positive correlation with CSF p-tau181 and t-tau, as well as with TNFR1 and ICAM-1. Further mediation analysis suggested that the impact of CSF α-synuclein on cognitive and executive functions is primarily mediated through CSF t-tau and p-tau181. Notably, CSF α-synuclein also influences CSF tau pathology through interactions with CSF TNFR1 and ICAM-1. These findings underscore the importance of monitoring CSF α-synuclein levels as a potential biomarker for identifying individuals at an increased risk of cognitive decline and dementia.

Changes in synaptic function and loss represent early and pivotal events in the development of dementia. For instance, in AD, early synaptic dysfunction is significantly associated with cognitive decline, a relationship that is more pronounced than the association with morphological changes [17]. α-synuclein serves as one of the markers of synaptic dysfunction and plays a crucial role in the process of cognitive decline. In DLB, the early accumulation of α-synuclein in the cortex distinguishes it from PD, underscoring the predominance of cognitive symptoms over motor symptoms [18]. The results indicated that higher levels of α-synuclein was associated with increased risk of developing into dementia, with a poor cognition and reduced executive function. This finding aligns with previous studies that have linked elevated levels of α-synuclein to cognitive decline and a reduced quality of life [19]. However, some studies have reported conflicting findings regarding CSF α-synuclein levels in dementia, observing them to be increased, decreased, or unchanged [20]. These discrepancies could stem from factors such as contamination (e.g., with blood), variations in CSF collection, processing, analysis, and the presence of co-pathologies.

Co-pathologies were commonly observed in patients with dementia, with their interactions potentially exacerbating the neurodegenerative process [12, 21]. The concurrent presence of α-synuclein and tau within the same regions had been shown to enhance their mutual propagation, leading to an accelerated disease progression [12–14]. While previous studies had identified a strong positive correlation between CSF α-synuclein and tau pathology [21–24], others had reported a negative correlation [25, 26]. GSEA analysis highlighted significant enrichment in tau-related pathways, specifically the MAPK cascades, in the α-synuclein-H group. Correlation analysis supported this for CSF α-synuclein was positively corelated with both CSF t-tau and p-tau181. Prior research suggested a direct interaction between α-synuclein and tau, contributing to their co-aggregation. Specifically, α-synuclein had been shown to induce tau aggregation [27–30], and tau can, in turn, accelerated α-synuclein fibrillization [13]. However, whether α-synuclein and tau pathology synergistically worsen neurodegeneration or if one predominates remains unclear. The results suggested that α-synuclein’s impact on cognition was primarily mediated through t-tau and p-tau181, as indicated by the significance of the ACME and the mediation proportions, despite the ADE not being significant. These mediation effects were also observed in executive function assessments. Clinical autopsy reports had indicated that age-related cognitive decline was primarily driven by neurofibrillary tangles rather than co-pathologies like α-synuclein [31]. Nonetheless, recent findings suggest that α-synuclein pathology alone could initiate dementia development, even without extensive cortical involvement in LBD [12]. Given the variability in α-synuclein propagation and distribution across different conditions, future research should concentrate on regional differences in synaptic degeneration linked to α-synuclein load. Such an approach will enhance the understanding of cognition’s vulnerability under various pathological conditions and offer insights into selective neuropathological progression in dementia.

Numerous studies have investigated the role of TNFRs in triggering or worsening neurodegeneration and cognitive decline [32, 33]. Previous studies have highlighted the involvement of TNFRs in the processing of amyloid precursor protein (APP) and the subsequent deposition of Aβ [32], and promotion on paired helical filament formation [34]. This study observed an upregulation in the immune response pathway in individuals with higher levels of α-synuclein. Moreover, CSF TNFR1 and ICAM-1 positively correlated with CSF α-synuclein, t-tau, and p-tau181. TNFR1 acts as a transmembrane receptor for TNFα [35] and is widely expressed in nearly all cell types. It is associated with the pro-inflammatory actions of tumor necrosis factors (TNF) [36]. Genetic deletion of TNFR1 results in reduced plaque deposition in the APP23 mouse model [37]. Previous studies have noted a marked increase in the expression of TNFR1 genes in the brains of AD patients compared to controls [38–40]. In CA1 pyramidal neurons, the TNF/TNFR1-mediated necroptosis pathway is significantly activated in AD-affected brains [38]. Certain inflammatory molecules indeed exhibit dual roles in both promoting and inhibiting inflammation [41, 42]. Low levels of soluble ICAM-1 (sICAM-1) have been shown to trigger the activation of NFκB and ERK, leading to the release of inflammatory cytokines such as macrophage inflammatory protein-1α, MIP-2, TNFα, IFNγ, and IL-6 [43, 44]. In contrast, high levels of sICAM-1 enhance endothelial migration and angiogenesis, competitively inhibit leukocyte-endothelial cell interactions, and promote the pro-repair activity of immune cells [45, 46]. Moreover, in the later stages of immune response, TNFα exerts an anti-inflammatory effect by inhibiting the production of IL-12 p40 in macrophages and dendritic cells through TNFR1 [47]. This dual functionality is influenced by several factors, including the activity of inflammatory signaling pathways, the types of cells involved, and the specific conditions of the inflammatory microenvironment. It is noteworthy that the focus of drug development for neurodegenerative diseases has significantly shifted. While in 2016, the majority of drugs (56%) targeted amyloid, the current research and development landscape has pivoted towards therapies focused on inflammation and metabolism, now comprising 68% of the field [48, 49]. This shift highlights a growing recognition of the pivotal role that neuroinflammation plays in neurodegenerative processes [50].

This study has several limitations. First, the mediation model employed in this observational study elucidates the relationships between CSF α-synuclein, inflammatory factors, and tau pathology but falls short of establishing causality. Second, the absence of molecular biological experiments constrains our capacity to validate hypotheses concerning the neural pathological processes associated with α-synuclein. Incorporating such experiments in future studies would provide more direct insights into the underlying mechanisms. Third, the diagnoses were predominantly based on self-reported medical histories, and the study did not differentiate between types of dementia. It is noteworthy that the impact of α-synuclein on cognitive progression might vary depending on the initial brain regions affected and the specific forms of dementia. Investigating the regional differences in α-synuclein accumulation and identifying the contributing neuropathological processes are essential. Such studies could shed light on the mechanisms of selective vulnerability and the paths of neuropathological progression in dementia.

## Conclusions

In summary, this study elucidates a significant link between elevated levels of CSF α-synuclein and cognitive decline, underscoring the pivotal roles of activated inflammatory pathways and tau pathology in this correlation. It underscores the value of monitoring CSF α-synuclein levels as a promising biomarker for pinpointing individuals at an increased risk of experiencing cognitive deterioration and developing dementia.

### Supplementary Information

**Additional file 1.** Supplementary description about cohort identification, the detection process of CSF α-synuclein, the optimal cut-off value of α-synuclein for the risk of dementia, and baseline CSF biomarker characteristics between cognitively unimpaired and cognitive impairment.

**Additional file 2.** Supplementary description about details of the linear mixed-effects model investigated the longitudinal changes in cognition and executive function, and Kaplan-Meier survival curves and Cox proportional hazards model for conversion rates of non-AD to AD.

## Data Availability

ADNI is available at http:// adni.loni.usc.edu.

## Abbreviations

AD: Alzheimer’s disease
Aβ: Amyloid-β
CSF: Cerebrospinal fluid
MCI: Mild cognitive impairment
PD: Parkinson’s disease
DLB: Dementia with Lewy bodies
LBD: Lewy Body disorders
α-synuclein-L: Lower level of α-synuclein
α-synuclein-H: Higher level of α-synuclein
GSEA: Gene set enrichment analysis
TNFR1: Tumor necrosis factor receptor 1
ICAM-1: Intercellular cell adhesion molecule-1
MMSE: Mini-Mental State Examination
MoCA: Montreal Cognitive Assessment
ADAS-cog: Alzheimer’s Disease Assessment Scale-cognitive
