# Supplementary material for: Cerebrospinal fluid α-synuclein adds the risk of cognitive decline and is associated with tau pathology among non-demented older adults
Source: Alzheimers Res Ther. 2024 May 10;16:103. doi: 10.1186/s13195-024-01463-2 (PMC11084056; doi:10.1186/s13195-024-01463-2)
Supplement: Supplementary file 1 — Additional file 1. Supplementary description about cohort identification, the detection process of CSF α-synuclein, the optimal cut-off value of α-synuclein for the risk of dementia, and baseline CSF biomarker characteristics between cognitively unimpaired and cognitive impairment. [file 13195_2024_1463_MOESM1_ESM.docx]

**1. Cohort identification, inclusion, and exclusion in ADNI database**

A total of 389 participants were recruited for the Alzheimer’s Disease Neuroimaging Initiative (ADNI-1) across more than 50 sites in the United States and Canada. The protocol for data collection and sharing was approved by the institutional review boards of all participating institutions, in line with the ethical guidelines outlined in the Declaration of Helsinki. All participants, or their guardians, provided written informed consent.

Participants, aged between 55 and 90, were classified as either cognitively normal or with mild cognitive impairment (MCI). Cognitively normal participants had Mini-Mental State Examination (MMSE) scores ranging from 24 to 30, a Clinical Dementia Rating (CDR) of 0, no reported memory complaints, and no significant impairments in cognitive functions or daily living activities. MCI participants also scored 24 to 30 on the MMSE but differed with a CDR of 0.5, validated memory complaints by an informant, and objective memory loss, not fulfilling the criteria for probable Alzheimer’s Disease (AD) as per NINCDS/ADRDA guidelines. A diagnosis of dementia during follow-up was made with a CDR score of 1.0 or greater, subjective cognitive complaints reported by the subject, their study partner, or a clinician, along with objective cognitive and daily living function impairments. The inclusion criteria required participants to undergo detection of cerebrospinal fluid (CSF) α-synuclein biomarkers, core AD CSF biomarkers (total tau [t-tau], phosphorylated tau [p-tau], and amyloid beta 42 [Aβ42]), and CSF inflammatory biomarkers. Individuals diagnosed with dementia at baseline or lacking annual neuropsychiatric assessments were excluded. Additionally, to account for potential contamination (e.g., with blood) that could result in aberrant α-synuclein levels, participants with α-synuclein levels more than 3 standard deviations (SD) above the mean at baseline were excluded. The mean α-synuclein level was determined to be 0.7987 ng/mL with a standard deviation (SD) of 1.01834. Therefore, the inclusion range for α-synuclein levels was calculated to be from -0.2196 ng/mL to approximately 3.8537 ng/mL. Consequently, 331 non-demented participants were included in the analysis.

Utilizing the surv_cutpoint function from the R package survminer, an optimal cut-off value of 0.68 ng/mL for α-synuclein was determined to assess the risk of dementia. Based on this value, participants were categorized into two groups: lower α-synuclein (α-synuclein-L, n= 245) and higher α-synuclein (α-synuclein-H, n= 86). The study spanned 2351 person-times over a follow-up period of up to 150 months, with annual assessments.

**2. The detection process of cerebrospinal fluid (CSF) α-synuclein in the ADNI database**

α-Synuclein, a neuronal protein consisting of 140 amino acids, plays a vital role in synaptic vesicle transport and dopamine regulation under physiological conditions [1, 2]. However, under pathological stress—such as inflammation, oxidative damage, or exposure to neurotoxins like 1-methyl-4-phenyl-1,2,3,6-tetrahydropyridine—α-synuclein's expression levels can increase, leading to its aggregation. This aggregation process, starting with soluble monomers forming oligomers and eventually insoluble fibrils, is implicated in neuronal damage and is a hallmark of synucleinopathies and also exsited in other neurodegenerative disorders [3]. The detection of α-synuclein in CSF employs a Luminex assay, leveraging microspheres coated with antibodies that bind to α-synuclein. This multi-step process includes reagent preparation, sample incubation, and subsequent detection phases, ultimately analyzed by the Luminex 200™ instrument. Notably, the detection antibody used is a biotinylated goat anti-human α-synuclein antibody (R&D systems, catalog # BAF1338). The primary focus of this detection is on all kinds of α-synuclein.

**Figure S1. The optimal cut-off value of α-synuclein for the risk of dementia**^a^

**
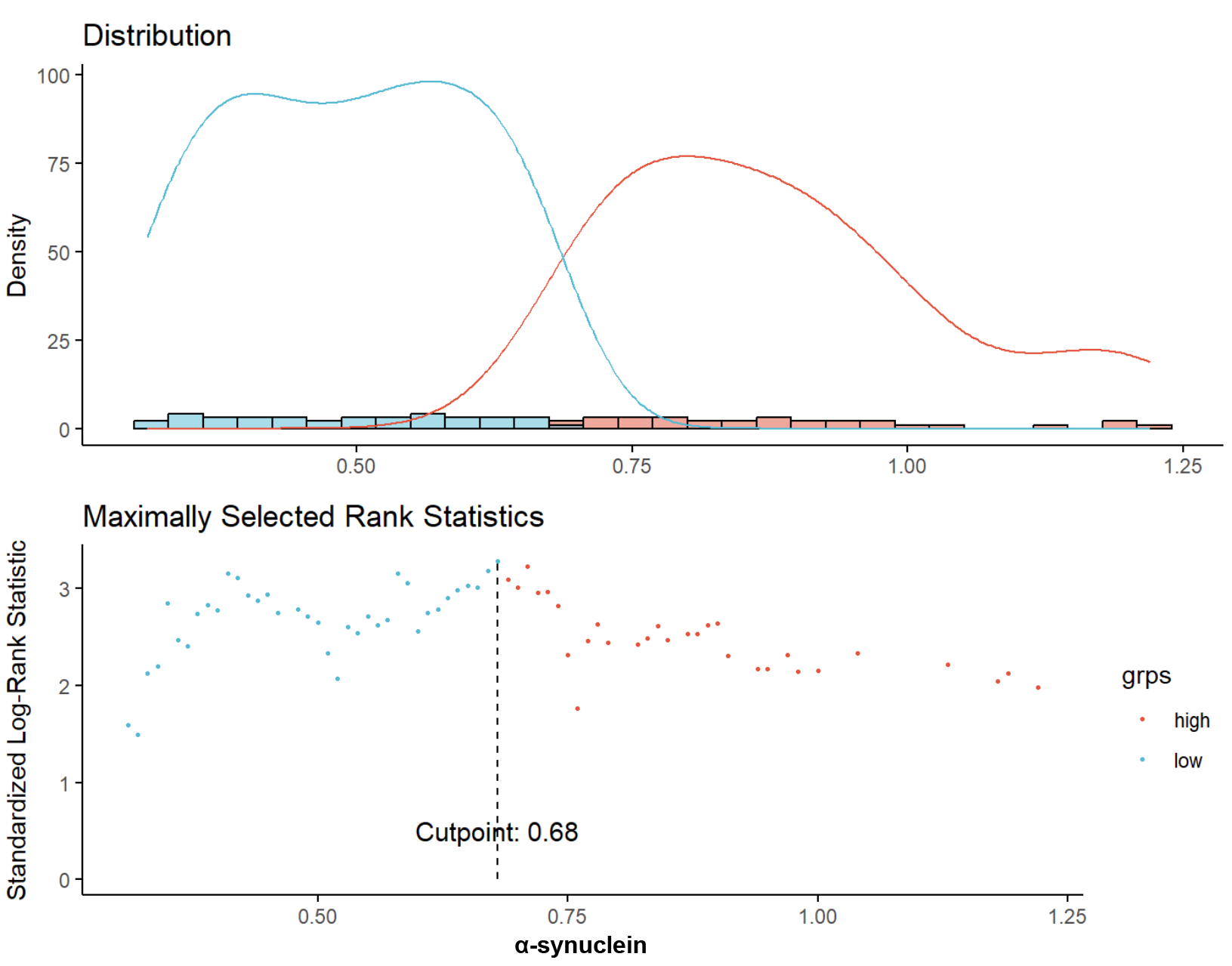
**

^a^Upon the surv_cutpoint function of the R package survminer in the R, the optimal cut-off value of α-synuclein for the risk of dementia is 0.68 ng/mL.

**Table S1. Baseline CSF biomarker characteristics between cognitively unimpaired and cognitive impairment in the non-demented population from the ADNI dataset.**

| Characteristics | | CU | MCI | p value |
| --- | --- | --- | --- | --- |
| N | | 104 | 227 |  |
| α-synuclein (ng/mL) | | 0.59 (0.54) | 0.70 (0.48) | < 0.0001^a^ |
| Aβ42 (pg/mL) | | 1265.00 (673.00) | 823.00 (477.00) | < 0.0001 |
| t-tau (pg/mL) | | 235.00 (82.60) | 321.00 (121.00) | < 0.0001 |
| p-tau181 (pg/mL) | | 21.90 (8.42) | 32.0 (14.20) | < 0.0001 |
| TNFR1 (pg/mL) | | 878.00 (219.00) | 882.00 (223.00) | 0.8770 |
| ICAM-1 (ng/mL) | | 362.00 (189.00) | 386.00 (206.00) | 0.5510 |
| ATN frame^b^ | A-T- | 44 | 29 | < 0.0001 |
|  | A+T- | 21 | 28 |  |
|  | A+T+ | 19 | 145 |  |
|  | A-T+ | 20 | 25 |  |

**Abbreviations:** CU: Cognitively unimpaired; MCI: Mild cognitive impairment; Aβ42: Amyloid beta 42; t-tau: Total tau; p-tau181: Phosphorylated tau at threonine 181; TNFR1: Tumor necrosis factor receptor 1; TNFR2: Tumor necrosis factor receptor 2.

^a^ Mann-Whitney U test

^b^ Utilizing the NIA-AA 2018 criteria, participants were categorized into four groups (A-T-, A+T-, A+T+, A-T+, with cutoffs: CSF Aβ42 < 976.6 pg/ml = A+; CSF p-tau181 >21.8 pg/ml = T+) [4].

**References:**

1. Morris HR, Spillantini MG, Sue CM, Williams-Gray CH: **The pathogenesis of Parkinson's disease**. *Lancet (London, England)* 2024, **403**(10423):293-304.

2. Kam TI, Mao X, Park H, Chou SC, Karuppagounder SS, Umanah GE, Yun SP, Brahmachari S, Panicker N, Chen R *et al*: **Poly(ADP-ribose) drives pathologic α-synuclein neurodegeneration in Parkinson's disease**. *Science (New York, NY)* 2018, **362**(6414).

3. Soto C: **α-Synuclein seed amplification technology for Parkinson's disease and related synucleinopathies**. *Trends in biotechnology* 2024.

4. Hansson O, Seibyl J, Stomrud E, Zetterberg H, Trojanowski JQ., Bittner T et al: ***CSF biomarkers of Alzheimer’s disease concord with amyloid-β PET and predict clinical progression: a study of fully automated immunoassays in BioFINDER and ADNI cohorts.*** *Alzheimers Dement*. 2018, **14**(1470–1481).
