# Supplementary material for: Cerebrospinal fluid α-synuclein adds the risk of cognitive decline and is associated with tau pathology among non-demented older adults
Source: Alzheimers Res Ther. 2024 May 10;16:103. doi: 10.1186/s13195-024-01463-2 (PMC11084056; doi:10.1186/s13195-024-01463-2)
Supplement: Supplementary file 2 — Additional file 2. Supplementary description about details of the linear mixed-effects model investigated the longitudinal changes in cognition and executive function, and Kaplan-Meier survival curves and Cox proportional hazards model for conversion rates of non-AD to AD. [file 13195_2024_1463_MOESM2_ESM.docx]

**Table S2. Association of α-synuclein-H group with changes in neuropsychiatric scales**^a^

| **Dependent variable** | **Mean difference (MD)**  **(α-synuclein-L vs**  **α-synuclein-H group)** | **SD** | **95%CI** | **p value** | **Mean difference (MD)**  **Time×α-synuclein-H group** | **SD** | **95%CI** | **p value** |
| --- | --- | --- | --- | --- | --- | --- | --- | --- |
| **Neuropsychiatric scale** | | | | | | | | |
| **MMSE** | -0.8308 | 0.4224 | -1.6587, 0.0029 | 0.0500 | -0.0024 | 0.0041 | -0.0104, 0.0056 | 0.5560 |
| **ADAS cog** | 1.0980 | 0.8500 | -0.5680, 2.7640 | 0.1971 | 0.0680 | 0.0172 | 0.0342, 0.1017 | <0.0001 |
| **ADNI-MEM** | -0.3322 | 0.1114 | -0.5505, -0.1139 | 0.0031 | 0.0022 | 0.0006 | 0.0010, 0.0034 | 0.0006 |
| **FAQ** | 2.4060 | 0.8449 | 0.7500, 4.0620 | 0.0047 | 0.0016 | 0.0071 | -0.0123, 0.0155 | 0.8204 |
| **ADNI-EF** | -0.3674 | 0.1255 | -0.6134, -0.1214 | 0.0037 | 0.0011 | 0.0009 | -0.0007, 0.0029 | 0.1967 |

^a^**Dependent variable:** MMSE, ADAS cog, ADNI-MEM, FAQ, ADNI-EF; Factor: α-synuclein-H group; Covariates: age, gender, education, *APOE ε4 genotype.*

**Abbreviations:** MMSE: Mini-Mental State Examination; ADNI-MEM: Alzheimer’s Disease Neuroimaging Initiative memory score; ADAS-cog 11: Alzheimer’s Disease Assessment Scale cognitive section 11-item; FAQ: Functional Activities Questionnaire; ADNI-EF: Alzheimer’s Disease Neuroimaging Initiative executive function score; CSF: Cerebrospinal Fluid; *APOE* ε4: apolipoprotein E type 4 allele.

**Figure S2. Kaplan–Meier survival curves and Cox proportional hazards model**^a^ **for conversion rates of non-AD to AD**^b^


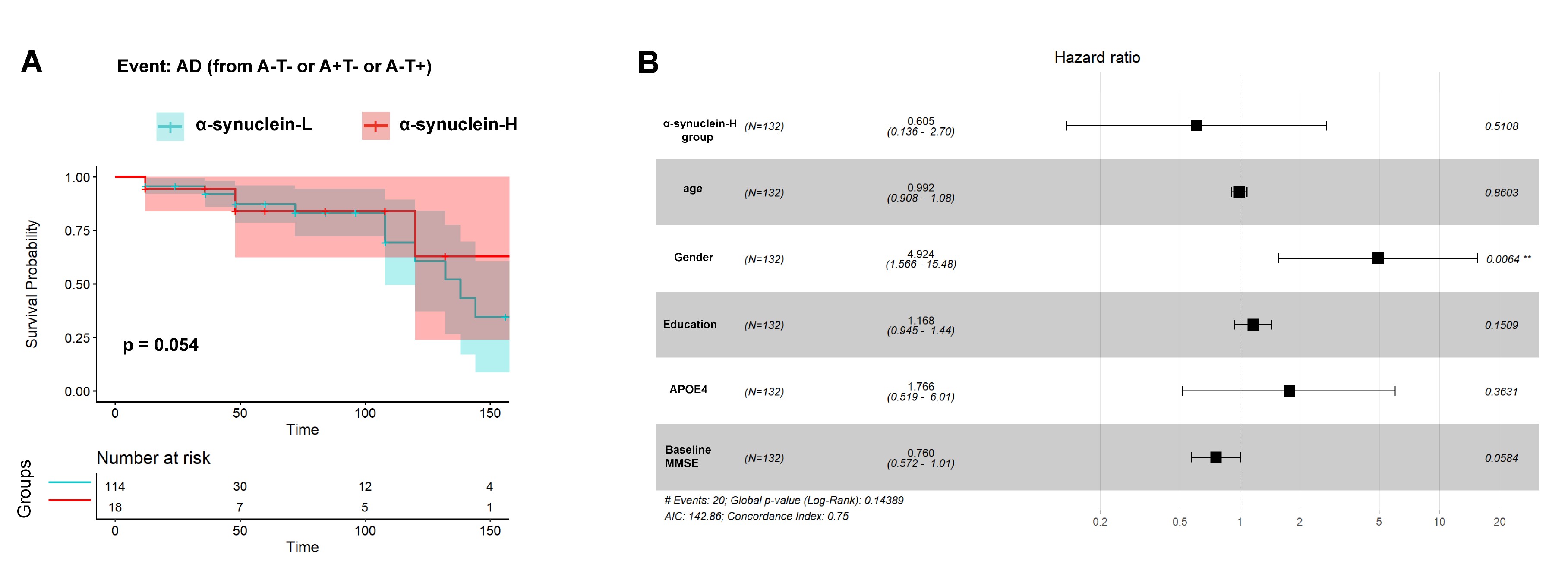


**Figure 2. Longitudinal effects of different α-synuclein groups on AD progression**. In the comparison of progression rates from non-AD to AD status, no significant difference was observed between the high α-synuclein (α-synuclein-H) group and the low α-synuclein (α-synuclein-L) group (A). Additionally, when adjustments were made for age, gender, education, and APOE ε4 genotype, the Cox proportional hazards model revealed that the α-synuclein-H group did not exhibit an increased risk of AD progression compared to the α-synuclein-L group (B)

**Abbreviations:** Alzheimer’s disease: AD; *APOE* ε4: apolipoprotein E type 4 allele; MMSE: Mini-Mental State Examination.

^a^covariates: age, gender, education, baseline MMSE, and *APOE ε4* genotype.

^b^Utilizing the NIA-AA 2018 criteria, participants were categorized into four groups (A-T-, A+T-, A+T+, A-T+, with cutoffs: CSF Aβ42 < 976.6 pg/mL = A+; CSF p-tau181 >21.8 pg/mL = T+) [1].

References:

1. Hansson O, Seibyl J, Stomrud E, Zetterberg H, Trojanowski JQ., Bittner T et al: ***CSF biomarkers of Alzheimer’s disease concord with amyloid-β PET and predict clinical progression: a study of fully automated immunoassays in BioFINDER and ADNI cohorts.*** *Alzheimers Dement*. 2018, **14**(1470–1481).
